# A new interstitial ostracod species of the genus *Paracobanocythere* from Vietnam, with mitochondrial *CO1* sequence data of three Asian species

**DOI:** 10.3897/zookeys.559.6751

**Published:** 2016-02-03

**Authors:** Hayato Tanaka, Le Doan Dung, Ryouichi Higashi, Akira Tsukagoshi

**Affiliations:** 1 Takehara Marine Science Station, Graduate School of Biosphere Science, Hiroshima University, 5-8-1 Minato-machi, Takehara, Hiroshima, 725-0024, Japan; 2 Environment and Energy Systems, Graduate School of Science and Technology, Shizuoka University, 836 Oya, Suruga-ku, Shizuoka, 422-8529, Japan; 3 Research Institute for Marine Fisheries (RIMF), 224 Le Lai, Hai Phong, Vietnam; 4 Fujieda Higashi High School, 1-7-1 Tennoucho, Fujieda, 426-8577, Japan

**Keywords:** DNA barcode, interstitial animals, meiofauna, Southeast Asia

## Abstract

This study is a first report of an interstitial ostracod from Southeast Asia. The ostracod species, *Paracobanocythere vietnamensis* sp. n., was found in the marine interstitial environment of Phu Quoc Island, Vietnam. Thus far, three species of this genus have been described. The morphology of the carapace as well as the appendages of this new species are quite similar to *Paracobanocythere hawaiiensis* and *Paracobanocythere watanabei*. However, we found that they could be easily distinguished according to the morphology of the male copulatory organ. Additionally, we estimated the evolutionary distances among these three species based on nucleotide and amino acid sequences of the mitochondrial *CO1* gene. Similar morphologies of carapaces and appendages, and relatively small evolutionary distances according to *CO1* between *Paracobanocythere vietnamensis* **sp. n.** and *Paracobanocythere watanabei* suggest that these two species are very closely related.

## Introduction

Ostracods are small bivalve crustaceans that inhabit various aquatic environments. They are one of the major constituents of the meiobenthos, especially interstitial animals inhabiting the pore space in sediment (Giere 2009). Interstitial ostracods are found in the Atlantic, Indian and Pacific Oceans (Rao 1972, Danielopol and Hartmann 1986). Although a number of taxonomic studies have been performed on extant and fossilized marine ostracods from the marginal sea located in Southeast Asia (e.g. Brady 1880, Müller 1906, Kornicker 1970, Keij 1974, 1975, Hanai et al. 1980, Tanaka et al. 2009), interstitial species have, thus far, not been reported.

This study is the first description of an interstitial ostracod species from Southeast Asia. The new species belongs to the genus *Paracobanocythere*, which shows typical features of interstitial taxa, including a small and dorso-ventrally depressed carapace inhabiting the interstices between grains of coarse sand (Gottwald 1983, Hartmann 1991, Higashi and Tsukagoshi 2011). Thus far, three species of this genus have been described; these include *Paracobanocythere hawaiiensis* Gottwald, 1983 (type species), from the island of O’ahu in Hawaii, as well as *Paracobanocythere grandis* Higashi & Tsukagoshi, 2011 and *Paracobanocythere watanabei* Higashi & Tsukagoshi, 2011, from the sand beach in Shizuoka Prefecture, on the Pacific coast of central Japan. Here, we describe a new species from Vietnam and supply DNA sequence data of the mitochondrial *cytochrome c oxidase subunit 1*
(CO1) gene from the three described Asian species.

## Materials and methods

**Figure 1.**
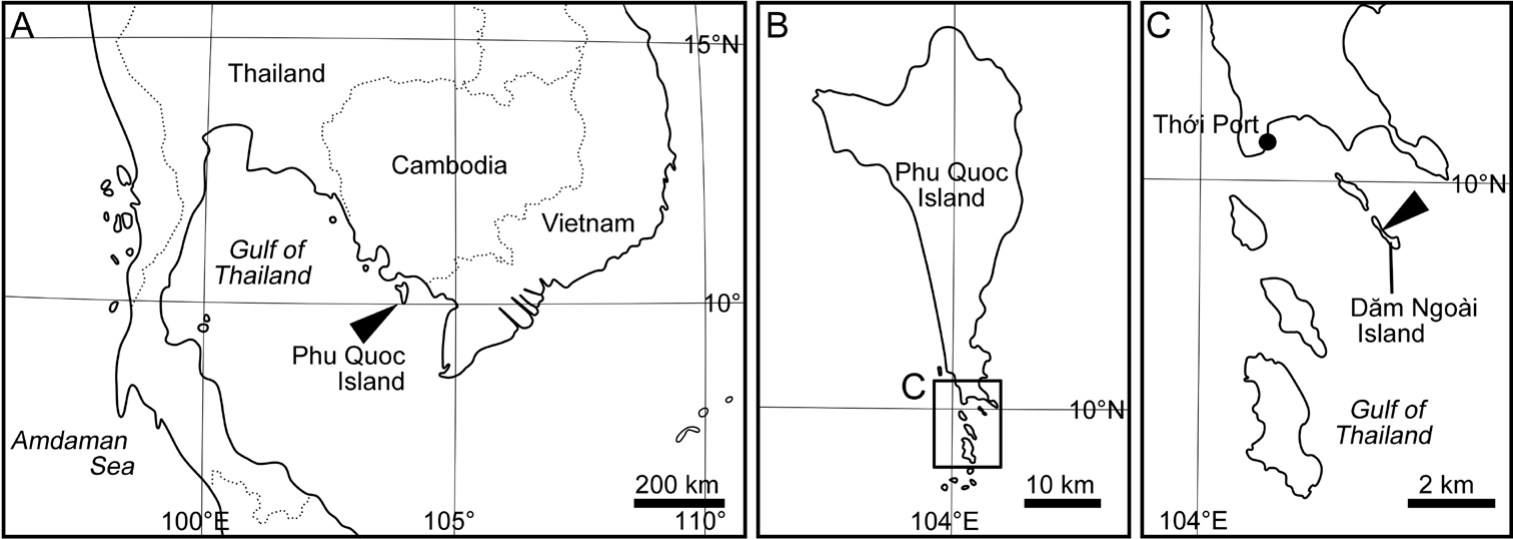
Map showing sampling locality of *Paracobanocythere vietnamensis* sp. n. **A** western part of Southeast Asia **B** Phu Quoc Island, Vietnam **C** The Dăm Ngoài Island. Arrowhead indicates the type locality.

Very coarse sand was collected from the Dăm Ngoài Island, in the Phu Quoc Marine Protected Area of Phu Quoc Island, from southwest Vietnam, 9°59'42"N, 104°02'17"E (Fig. 1) approximately 10 cm below the shoreline sand surface at low tide. The samples were washed five times in a bucket with sea water, and the supernatant was strained through a mesh with a 40-µm pore size. The living specimens were isolated from the deposits using a stereo-binocular microscope (SZ-60, OLYMPUS, Japan). The collected specimens were fixed in 80% ethanol and preserved at room temperature for description and DNA extraction. The soft parts of the organisms were separated from the valves and dissected using fine needles. The valves were preserved on a cardboard cell slide and the soft parts mounted in a gum-chloral medium, Neo-Shigaral (Shiga Konchu Fukyusha, Japan), on glass slides using a stereo-binocular microscope. The specimens were then observed and sketched using a transmitted-light binocular microscope (BX-53, OLYMPUS, Japan) with a differential interference contrast system and a camera lucida. The valves were washed with distilled water and gold-coated by an Ion sputtering device (JFC-1100, JEOL, Japan). The materials were then observed by SEM (JSM-6510LV, JEOL, Japan).

The type series was deposited in the collection of the National Museum of Nature and Science, Tokyo (NSMT), with the prefix ‘NSMT-Cr’.

### DNA experiment and analyses

The specimens of both *Paracobanocythere grandis* and *Paracobanocythere watanabei* used in DNA extractions were collected from the type localities (See Higashi and Tsukagoshi 2011, fig. 1): Mochimune Beach (34°55'04"N, 138°21'43"E), Shizuoka, central Japan, on 1 Aug. 2015 and Oura Beach (34°40'05"N, 138°56'28"E), Shizuoka, central Japan, on 30 Sep. 2012. Details of the specimens from the three species used for DNA experiment are found in Table 1. Total DNA extraction was performed using the DNeasy Blood and Tissue Kit (Qiagen, USA) following the manufacturer’s protocol, except that the elution volume used was changed to 100 µl. To prepare samples for DNA extraction individuals were dissected as follows: the carapace and gut content was removed before DNA extraction, and after the protein digestion step the chitinous soft parts were retrieved from the microtube and mounted in the same manner as method of dissected soft parts samples. The valves and chitinous soft parts were preserved as morphological voucher specimens, and deposited in the NSMT with serial numbers.

**Table 1.** List of *CO1* sequences and vouchers for the three *Paracobanocythere* species.

Species	Specimen catalog number	GenBank number
*Paracobanocythere vietnamensis* sp. n.	NSMT-Cr 24323 (paratype)	LC101962
*Paracobanocythere vietnamensis* sp. n.	NSMT-Cr 24324 (paratype)	LC101963
*Paracobanocythere vietnamensis* sp. n.	NSMT-Cr 24325 (paratype)	LC101964
*Paracobanocythere grandis*	NSMT-Cr 24335(topotype)	LC101965
*Paracobanocythere grandis*	NSMT-Cr 24336(topotype)	LC101966
*Paracobanocythere grandis*	NSMT-Cr 24337(topotype)	LC101967
*Paracobanocythere watanabei*	NSMT-Cr 24338(topotype)	LC101968
*Paracobanocythere watanabei*	NSMT-Cr 24339(topotype)	LC101969
*Paracobanocythere watanabei*	NSMT-Cr 24340(topotype)	LC101970

Partial sequences of the mitochondrial *CO1* gene were PCR amplified using the following primers: a degenerate forward primer (COIO_F 5’- CNACNAAYCAYAARGATATTGG -3’) designed in this study and the universal reverse primer HCO2198 (Folmer et al. 1994). This region is the most commonly used for DNA barcoding to identify animals (Hebert et al. 2003, Bucklin et al. 2011). The 25 µl reaction contained 0.125 µl of *TaKaRa Ex Taq* HS (TAKARA BIO Inc., Japan), 2.5 µl of 10×*Ex Taq* buffer, 2 µl of dNTP mix, 2 µl of each primer (5 pmoles each), 2 µl of template DNA, and 14.375 µl sterilized distilled water. The PCR protocol consisted of an initial denaturation step at 95 °C for 2 min, followed by 40 cycles of denaturation at 95 °C for 20 s, annealing at 40 °C for 30 s, extension at 72 °C for 1 min, and a final extension at 72 °C for 10 min. Quantity and length of the PCR products were checked by 1% agarose S (Nippon Gene, Japan) gel electrophoresis and stained with ethidium bromide. The products were purified for sequencing using a FastGene Gel/PCR Extraction Kit (NIPPON Genetics Co,Ltd, Japan), according to the manufacturer’s protocol. Sequencing (of both the forward and reverse reads) was performed by the Macrogen Japan Corp. (Tokyo) with the same primers as were used for PCR amplification. A homology search of *CO1* sequences was performed by BLAST (Altschul et al. 1990, 1997) with the discontiguous megablast program from the National Center for Biotechnology Information (NCBI, http://blast.ncbi.nlm.nih.gov/Blast.cgi).

The *CO1* sequences were converted to amino acids based on the invertebrate mitochondrial genetic codon using MEGA6 (Tamura et al. 2013). The evolutionary distances of both the nucleotide and amino acid sequences were estimated with MEGA6 (Tamura et al. 2013) using Kimura’s two parameter model (Kimura 1980) and the Poisson model (Nei and Kumar 2000), respectively. Standard error estimates were obtained by a bootstrap procedure (1000 replicates).

## Taxonomy

### Order Podocopida Sars, 1866 Superfamily Cytheroidea Baird, 1850 Family Cobanocytheridae Schornikov, 1975 Genus *Paracobanocythere* Gottwald, 1983

#### Paracobanocythere vietnamensis

Taxon classificationAnimaliaPodocopidaCobanocytheridae

Tanaka & Le sp. n.

http://zoobank.org/EFD9A861-5477-488C-BC70-4B6861FFEB86

##### Type series.

Holotype: adult male (NSMT-Cr 24314), right valve length 323 µm, height 107 µm, left valve length 337 µm, height 111 µm, soft parts mounted on a slide and valves preserved in a cardboard cell slide. Paratypes: 11 adult males (NSMT-Cr 24315–24325) and 9 adult females (NSMT-Cr 24326–24334). All specimens were collected by Hayato Tanaka on 21 November 2014.

##### Type locality.

The holotype specimen was collected from Dăm Ngoài Island, Phu Quoc Marine Protected Area in Phu Quoc Island, the southwest Vietnam, 9°59'42"N, 104°02'17"E (Fig. 1C); in an interstitial environment at 10 cm below the shoreline sand surface. The substrate consisted mainly of very coarse sand (median grain size is about 2 mm).

##### Diagnosis.

Carapace elongate in lateral view and depressed dorsoventrally. Anterior and posterior margins rounded. Carapace surface smooth but with small granular texture visible at high magnification. Sieve-type normal pores with recessed sieve plates and thick rims on carapace surface. Left hemipenis bearing one additional pincer-like structure and one hooked process.

##### Description of adult male.

**Figure 2.**
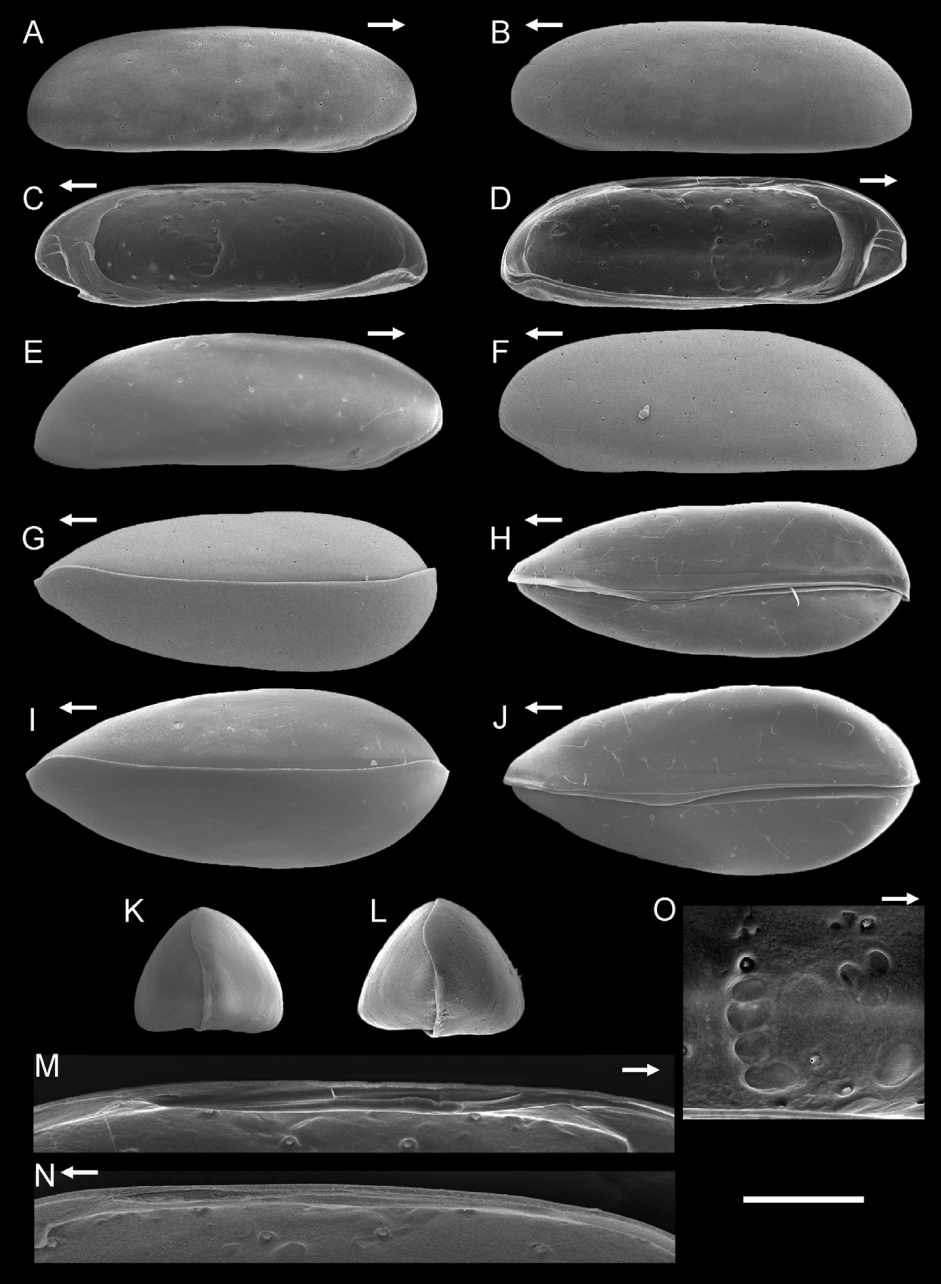
SEM images of valves and carapace of *Paracobanocythere vietnamensis* sp. n. **A** and **B** male paratype (NSMT-Cr 24315) **C, D, M–O** male, paratype (NSMT-Cr 24316) **E** and **F** female, paratype (NSMT-Cr 24327) **G** male, paratype (NSMT-Cr 24317) **H** male paratype (NSMT-Cr 24318) **I** female, paratype (NSMT-Cr 24328) **J** female, paratype (NSMT-Cr 24329) **K** male, paratype (NSMT-Cr 24319) **L** female, paratype (NSMT-Cr 24330). **A** right external lateral view **B** left external lateral view **C** right internal lateral view, anteroventral margin is slightly damaged **D** left internal lateral view **E** right external lateral view **F** left external lateral view **G** dorsal view **H** ventral view **I** dorsal view **J** ventral view **K** anterior view **L** anterior view **M** hingement part of left valve **N** hingement part of right valve **O** adductor muscle scars of left valve. Scale bar indicates 100 µm (**A–N**) and 40 µm (**O**). Arrows indicate anterior direction.

**Figure 3.**
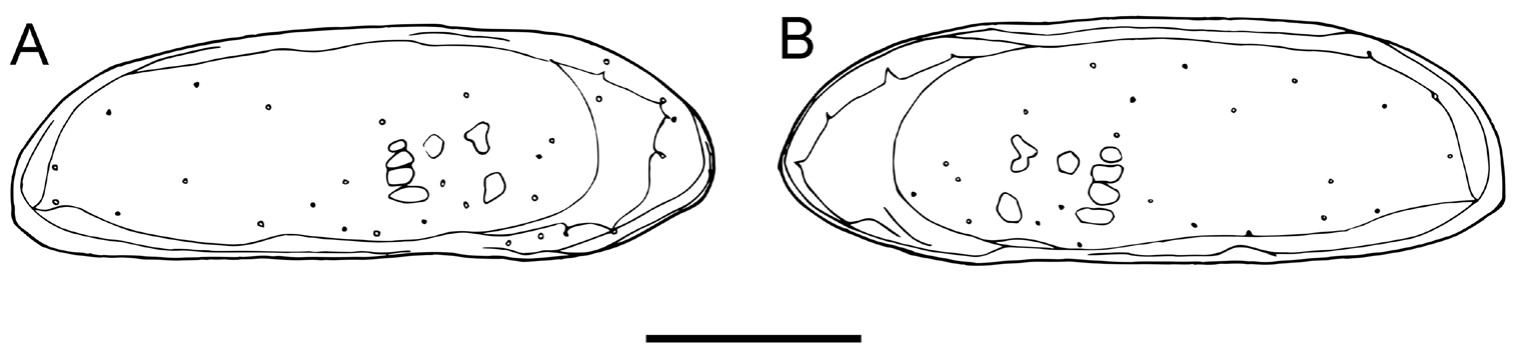
Valves of *Paracobanocythere vietnamensis* sp. n. Male, holotype (NSMT-Cr 24314). **A** left internal lateral view **B** right internal lateral view. Scale bar indicates 100 µm.

**Figure 4.**
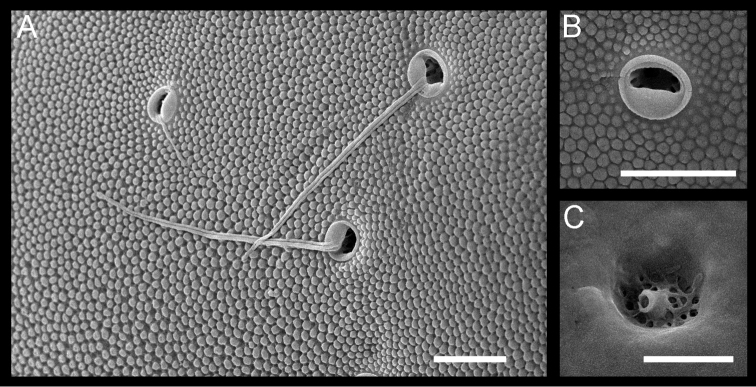
SEM images of the detailed structure of *Paracobanocythere vietnamensis* sp. n. **A** and **B** male paratype (NSMT-Cr 24315) **C** male paratype (NSMT-Cr 24316) **A** three pore systems and small granular texture in external lateral view **B** sieve type normal pore with recessed sieve plate and thick rim **C** internal view of sieve plate of sieve type normal pore. Scale bars indicate 5 µm.

Carapace (Figs 2A–D, G–H, K, M–O; 3; 4). Length and height of left valves greater than those of right valve (Table 2). Carapace elongated in lateral view and depressed dorso-ventrally. In anterior view, carapace rounded triangular (Fig. 2K). Left valve slightly overlapping right valve along anterior and posterior margins. Anterior and posterior margin rounded in lateral view. Marginal infold broad along anterior margin and narrow in posterior area (Fig. 2C, D). Anterior vestibulum occupying most of area in marginal infold, containing five and six marginal pore canals in left and right valve, respectively (Fig. 3). In both valves a thick, irregular ridge runs diagonally upward across the anterior infold, and three short buttress-like ridges or wrinkles run anteriorly from the upper part of this ridge for additional strength. Adductor muscle scar pattern consisting of row of four elongate closely spaced scars and three frontal scars (Figs 2O; 3). Carapace surface with faint granular texture visible at high magnification, resulting from close-packed, tiny tubercules (Fig. 4A). Sieve type normal pores with recessed sieve plates and thick rims on carapace surface (Fig. 4). Pore systems with one bristle. Hingement adont type (Fig. 2M, N). Color translucent white; living individuals have brown granular patterns.

**Table 2.** Dimensions of valves of *Paracobanocythere vietnamensis* sp. n.

			Length (µm)			Height (µm)	
		Mean	Observed range	N	Mean	Observed range	N
Male	Right valve	332	322–338	7	107	100–111	7
	Left valve	337	325–347	7	111	105–117	7
Female	Right valve	349	342–359	5	115	113–119	5
	Left valve	354	346–361	5	119	115–120	5

**Figure 5.**
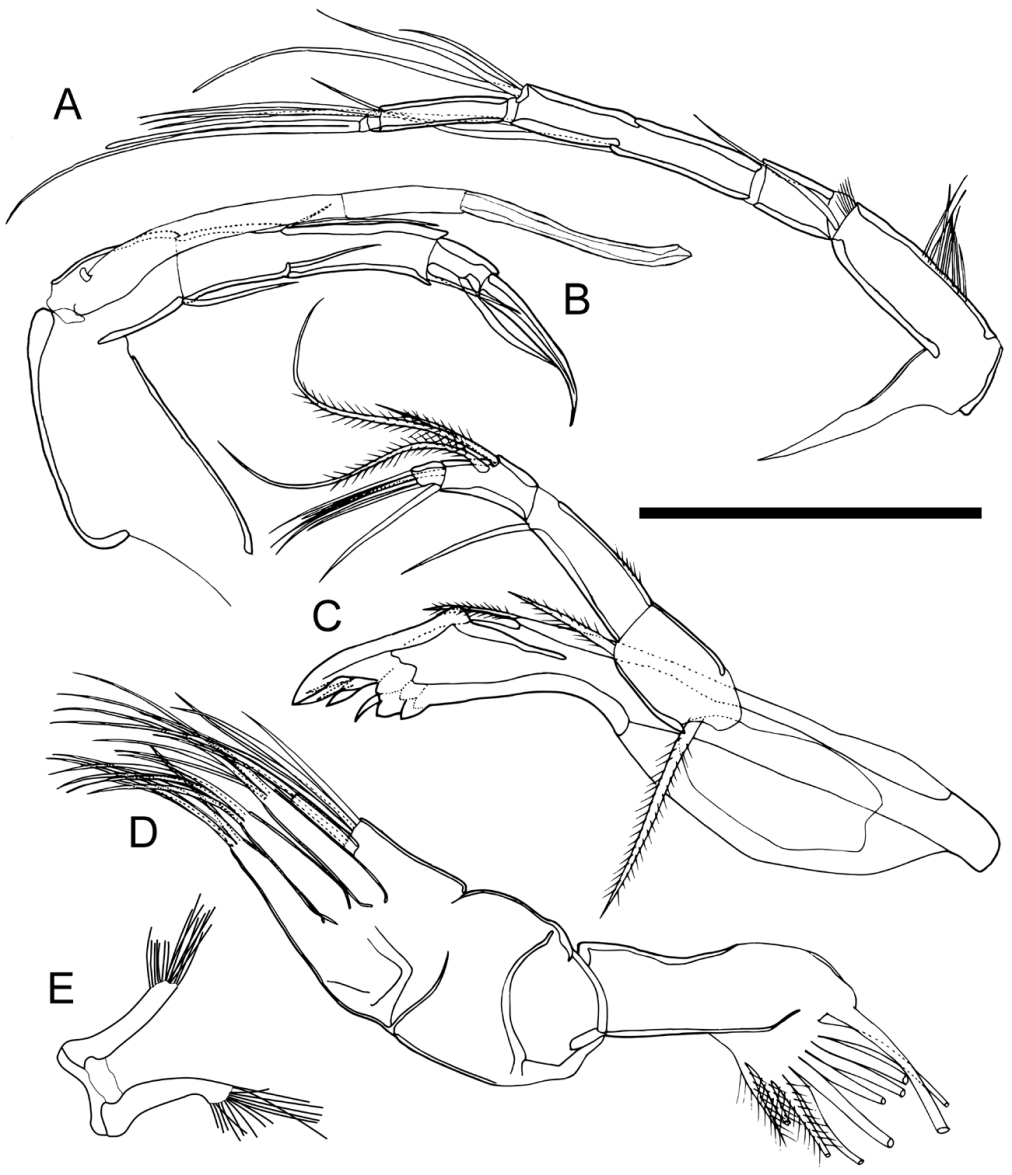
Appendages of *Paracobanocythere vietnamensis* sp. n. Male, holotype (NSMT-Cr 24314). **A** antennula **B** antenna **C** mandibula **D** maxillula **E** brush-shaped organ. Scale bar indicates 50 µm (**A–D**) and 25 µm (**E**).

Antennula (Fig. 5A). Consists of six articulated podomeres, of which fourth and fifth are incompletely separated. First podomere bare. Second podomere about two and a half times as long as first podomere, with one long posterodistal seta and short setulae on distal end and eight coarser setulae on anterior margin. Third podomere same length as first podomere and bare. Fourth podomeres twice as long, with one long posterodistal seta. Fifth podomere almost as long, with three anterodistal setae of staggered lengths and one posterodistal seta. Sixth podomere long, slender, with three long anterodistal setae and long distal seta fused at its base with distal aesthetasc.

Antenna (Fig. 5B). Four articulated podomeres. First podomere (basis) bare and slightly triangular, tapering distally, with a long, thick, three-segmented exopodite (spinneret seta) reaching beyond distal claws. Second (first endopodial) podomere with one short seta on posterodistal end. Third podomere with one short and one medium anterodistal setae, one short posteromedial seta, and one short posterodistal seta. Fourth podomere with one long stout posterodistal seta and one curved stout distal claw.

Mandibula (Fig. 5C). Coxa with one short setulous seta on anterior margin. Coxal endite consisting of seven teeth, two short setae and one short claw-like seta. Palp consisting of four indistinct podomeres. Basis with one long setulous seta (exopodite) near proximal end and medium setulous seta on posterodistal end. First podomere of endopodite about one and a half times as long as basis, with one medium anterodistal seta and setulae on anterior margin. Second podomere half as long as first podomere, with two long and one medium setulous setae on middle of anterior margin, one medium mediodistal seta, and one medium posterodistal seta. Third podomere small, with four medium setae on distal end.

Maxillula (Fig. 5D). Thin branchial plate (exopodite) with ten plumose setae. Basal podomere with one palp (endopodite) and three endites. Palp consisting of two distinct podomeres: first podomere with five long setae on distal end; second podomere two-thirds as long as first podomere, with one long and one medium setae on distal end. Outer two endites with five setae, and posteriormost one with four setae.

Male brush-shaped organ (Fig. 5E). Consisting of two branches (right and left) each with 16 setae on distal margin.

**Figure 6.**
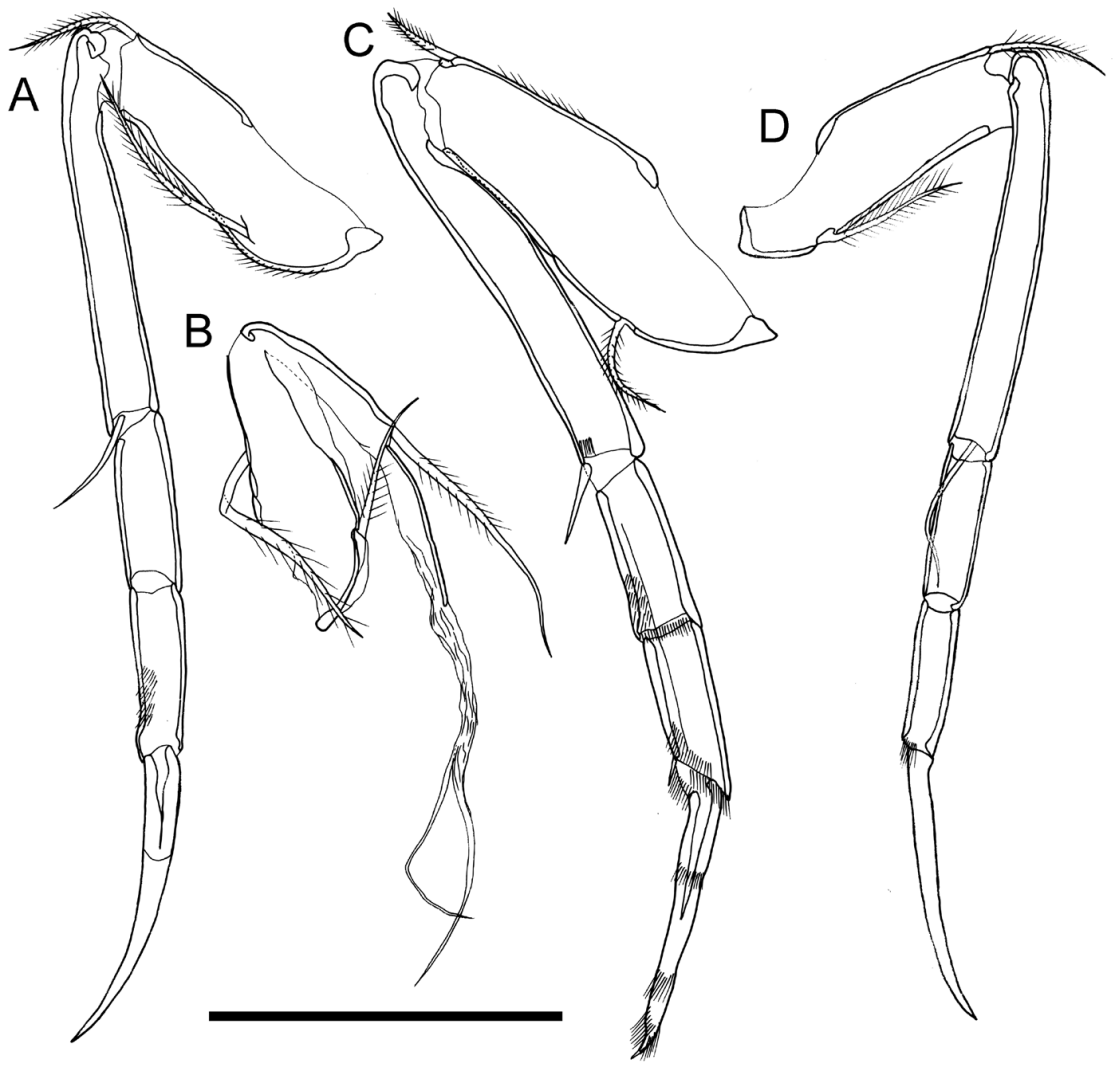
Appendages of *Paracobanocythere vietnamensis* sp. n. **A** and **C** male paratype (NSMT-Cr 24320) **B** male holotype (NSMT-Cr 24314) **D** female paratype (NSMT-Cr 24326) **A** fifth limb **B** sixth limb **C** seventh limb **D** sixth limb. Scale bar indicates 50 µm.

Fifth limb (Fig. 6A). Four articulated podomeres; two distal podomeres and claw somewhat thickened. First podomere with one medium setulous anterodistal seta, one long setulous posteroproximal seta and setulae along both margins. Second podomere five-fourths as long as first podomere, with one short anterodistal seta. Third podomere bare and half length of second podomere. Fourth podomere same length as third podomere with rows of setulae on anterior surface and one stout distal claw.

Sixth limb (Fig. 6B). Three podomeres, of which two are flimsy and weakly developed. First podomere with one medium setulous seta on middle of anterior margin and one long setulous seta on middle of posterior margin. Second podomere two-thirds as long as first podomere, posterior margin flabby, with one long setulous seta on antero-distal end. Basal part of third podomere same length as second podomere, with flabby elongated sheet distally and two weakly developed long branches.

Seventh limb (Fig. 6C).Four articulated podomeres, all very large. First podomere with one short setulous seta on antero-distal end, one medium setulous seta on near postero-proximal part, and setulae along anterior margin. Second podomere five-fourths as long as first podomere, with one short seta and row of setulae on antero-distal end. Third podomere one-third as long as second podomere, with rows of setulae on anterior surface and distal margin. Fourth podomere same length as third podomere, with rows of setulae on anterior surface and distal margin, with one stout, club-shaped distal claw with rows of setulae on middle part, near distal part, and around distal end.

**Figure 7.**
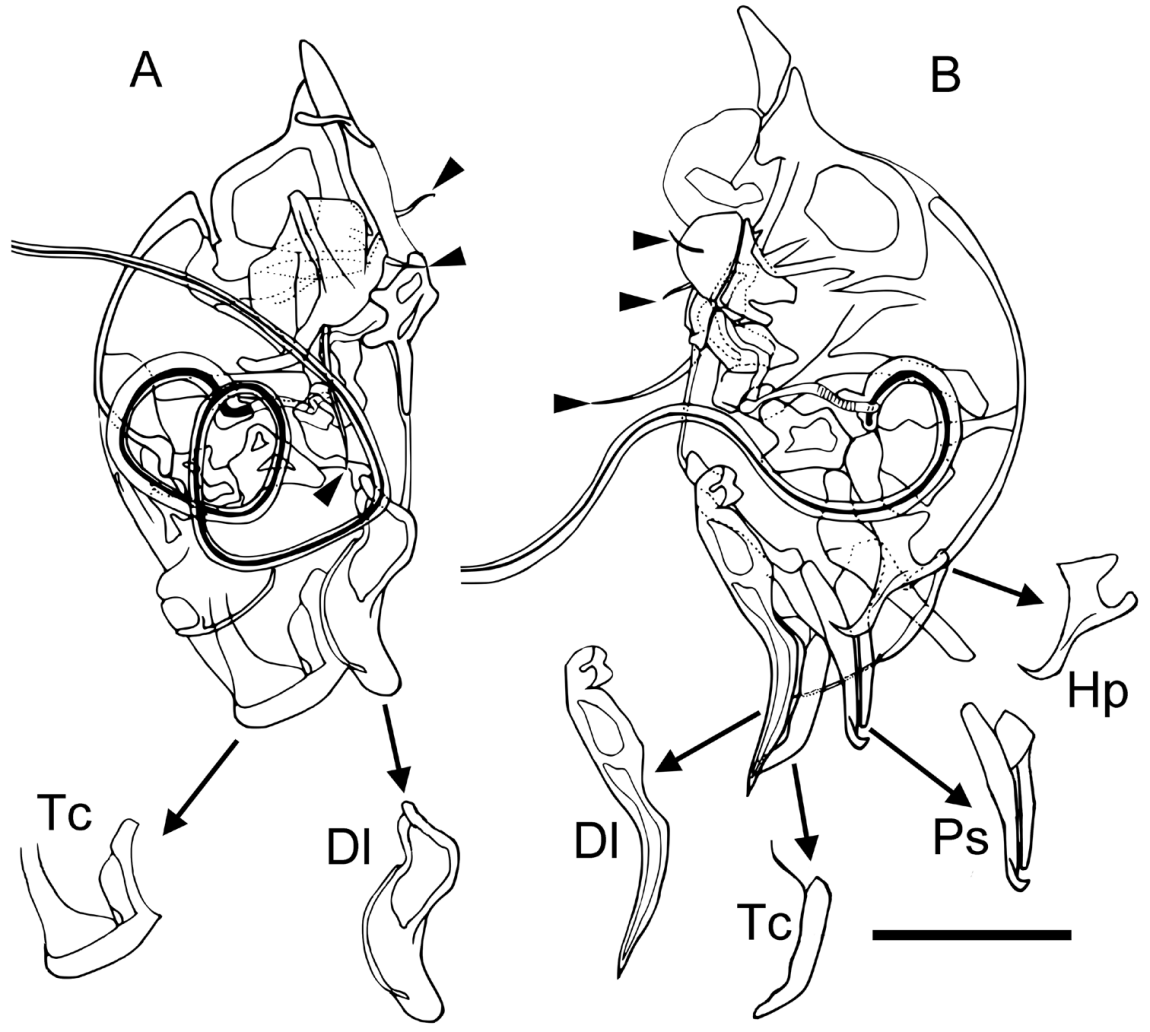
Copulatory organ of *Paracobanocythere vietnamensis* sp. n. Male, paratype (NSMT-Cr 24320) **A** internal view of right organ **B** internal view of left organ. Abbreviations: Dl distal lobe
Hp hooked process
Ps pincer-like structure
Tc tip of capsule. Scale bar indicates 50 µm.

Male copulatory organ (Fig. 7). Copulatory duct very long, more than length of capsule. Tip of capsule (Tc) and distal lobe (Dl) asymmetric in right and left hemipenes. Right hemipenis (Fig. 7A): Tc almost square distal part with rounded corner; Dl finger-shaped, bending ventrally at half. Left hemipenis (Fig. 7B): Tc slender, bending ventrally near the tip; Dl acute-angle triangular, curving ventrally at half; additional pincer-like structure (Ps) and hooked process (Hp) exist. Both hemipenes bearing one long and two short setae on ventral margin (vestigial furca).

Eye. Present.

##### Description of adult female.

Carapace (Fig. 2E, F, I, J, L). Both left and right valve of female slightly greater than valves of male (Table 2, Fig. 9). In dorsal view, width of carapace slightly greater than that of male (Fig. 2I, J). Anterior and posterior margins more tapered rather than those of male (Fig. 2E, F).

Sixth limb (Fig. 6D). Four articulated podomeres with slender, more normal proportions than the male limb. First podomere with one medium setulous anterodistal seta and one medium setulous posteroproximal seta. Second podomere four-thirds as long as first podomere, with one short anterodistal seta. Third podomere bare and half as long as first podomere. Fourth podomere same length as third podomere, with row of setulae on anterior distal surface, with one tapering distal claw.

**Figure 8.**
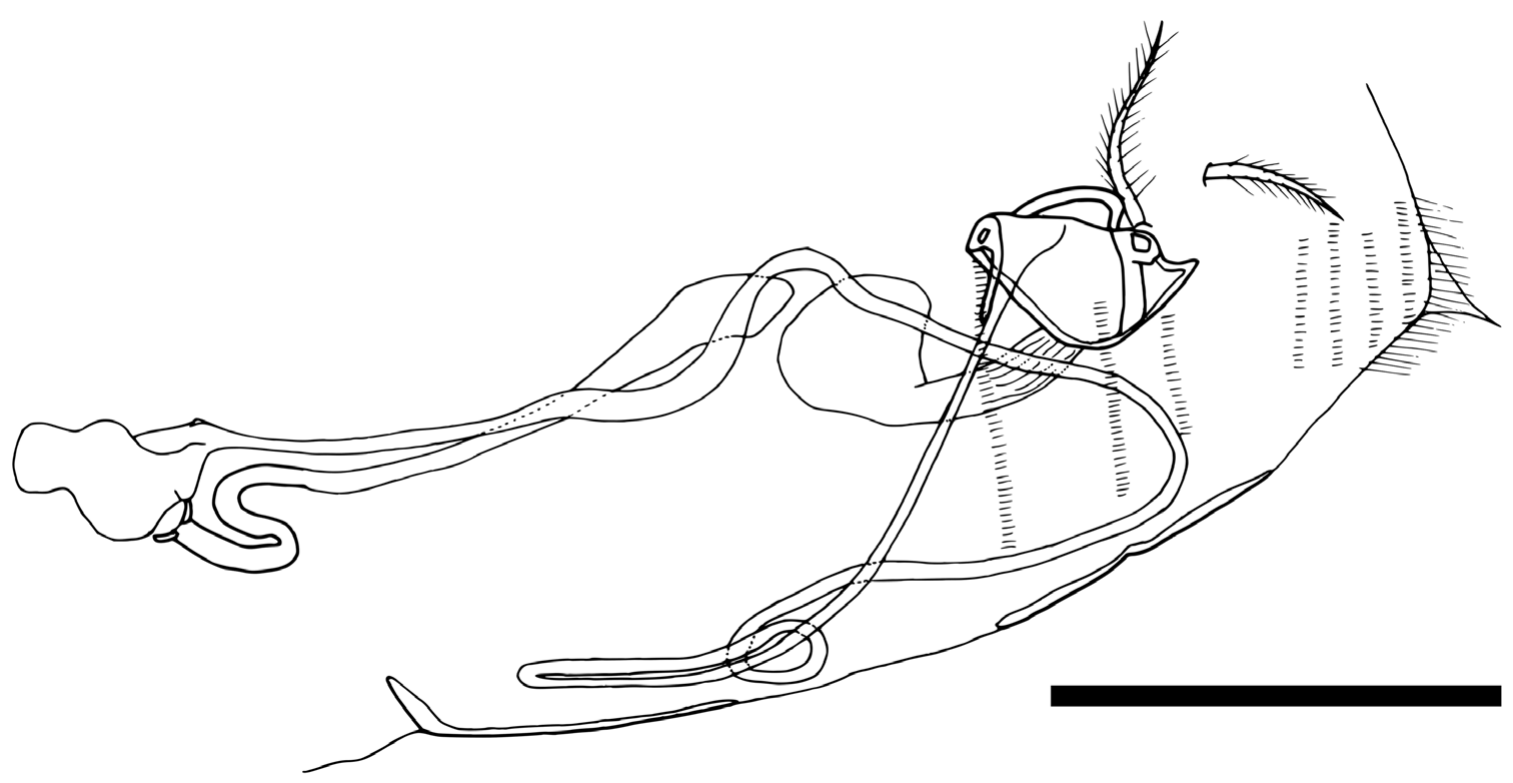
Posterior body and genitalia of *Paracobanocythere vietnamensis* sp. n. Female, paratype (NSMT-Cr 24326). Scale bar indicates 50 µm.

Posterior part of body and female genitalia (Fig. 8). Sclerotized framework of paired genital openings trapezoidal in shape. Spermathecal duct very long, connecting with genital opening and receptaculum seminis. Two setulose setae (vestigial caudal rami) situated near each genital opening. Five rows of tiny setulae on abdominal end.

##### Dimensions.

**Figure 9.**
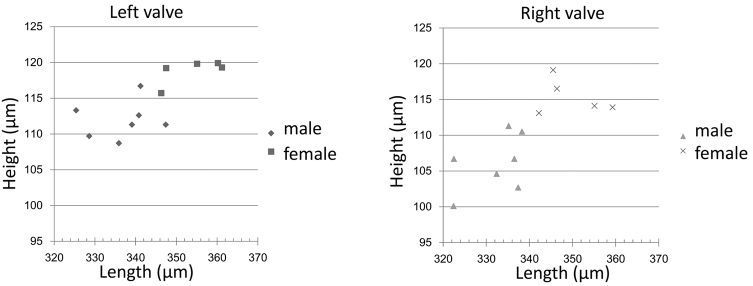
Scatter plots of valves of *Paracobanocythere vietnamensis* sp. n.

See Table 2 and Fig. 9.

##### Occurrence.

So far known only from type locality.

##### Etymology.

Named in recognition of this being the first record of *Paracobanocythere* from Vietnam.

##### Remarks.

*Paracobanocythere grandis* and other three species including *Paracobanocythere vietnamensis* sp. n. are easily distinguishable by the length of carapace (Table 3). While *Paracobanocythere grandis* has an exceptionally large carapace (approximately 500 µm) for this genus (Higashi and Tsukagoshi 2011), those of the other three species are relatively smaller (roughly 300 µm) (Gottwald 1983, Higashi and Tsukagoshi 2011). Furthermore, the female carapace larger than that of the male in *Paracobanocythere grandis*, and that is opposite to the status in the other three species (Table 3). The carapace shape as well as appendage morphologies including chaetotaxy and the number of podomeres, of *Paracobanocythere vietnamensis* sp. n. are quite similar to those in *Paracobanocythere hawaiiensis* and *Paracobanocythere watanabei* (Table 3). One slight difference is found in the chetotaxy of the sixth limb of male, i.e., the first podomere lacks the one short seta on antero-distal end, which is present in *Paracobanocythere hawaiiensis* and *Paracobanocytherewatanabei* but absent in *Paracobanocythere vietnamensis* sp. n. Moreover, the faintly granular texture of carapace surface of *Paracobanocythere vietnamensis* sp. n. has never been reported from *Paracobanocythere hawaiiensis* and *Paracobanocythere watanabei*. As for the morphology of the male coplatory organ, the new species can be easily distinguished from these two species. Specifically, the left hemipenis of *Paracobanocythere vietnamensis* sp. n. possesses Ps and Hp, whereas these structures are not observed in the original description of either of the two other species (Table 3).

**Table 3.** Morphological difference among four species of *Paracobanocythere*.

Character	*Paracobanocythere hawaiiensis*	*Paracobanocythere grandis*	*Paracobanocythere vietnamensis* sp. n.	*Paracobanocythere watanabei*
Male				
Carapace, length (left; right) [µm]	256–290	497–519; 481–503	325–347; 322–338	240–254; 236–250
height (left; right) [µm]	84–97	166–175; 157–165	105–117; 100–111	67–82; 72–80
granular texture on surface	–	–	present	–
Antennula, one seta on middle of anterior margin of fourth podomere	present	present	absent	absent
Mandibula, one seta on antero-distal end of basis	absent	present	present	present
Maxillula, seta number on endites (anterior; middle; posterior)	5 to 6	(6; 5; 4)	(5; 5; 4)	(5; 5; 4)
Sixth limb, one short seta on antero-distal end of 1st podomere	present	present	absent	present
third podomere	weakly developed	clearly segmented	weakly developed	clearly segmented
Seventh limb, proximal spines and hook-shaped structure on distal claw	absent	presnt	absent	absent
Brush-shaped organ, seta number	12	16	16	16
Copulatory organ, pincer-like structure and hooked process on left hemipenis	absent	absent	present	absent
Female				
Carapace, length (left; right) [µm]	260–344	466–486; 457–471	346–361; 342–359	252–266; 246–259
height (left; right) [µm]	92–117	162–171; 151–158	115–120; 113–119	81–89; 81–86
length and height larger than male	no	yes	no	no

### Evolutionary distances of nucleotide and amino acid sequences among three Asian species

The *CO1* sequences from *Paracobanocythere vietnamensis* sp. n., *Paracobanocythere watanabei* and *Paracobanocythere grandis* were obtained in this study. The lengths of the *CO1* barcoding region were 661 bp and the alignment of each sequence contained no indels. From this barcoding region, the first nucleotide was removed as it was not a complete codon, and the remaining 660 bp of the aligned sequence were translated into amino acid sequences. The evolutionary distances of both the nucleotide and amino acid sequences are shown in Table 4. The distances between *Paracobanocythere vietnamensis* sp. n. and *Paracobanocythere watanabei* are the least since this is the most closely related pair according to both the nucleotide and amino acid sequences.

**Table 4.** Evolutionary distances of *CO1* among three Asian species of *Paracobanocythere*. Standard error estimate are shown above the diagonal and were obtained by a bootstrap procedure (1000 replicates). **A** the result of nucleotide sequences with Kimura’s two parameter model **B** the result of amino acid sequences with Poisson model.

A		1	2	3
	1 *Paracobanocythere vietnamensis* sp. n.		0,03	0,03
	2 *Paracobanocythere watanabei*	0,31		0,03
	3 *Paracobanocythere grandis*	0,41	0,37	
B		1	2	3
	1 *Paracobanocythere vietnamensis* sp. n.		0,02	0,04
	2 *Paracobanocythere watanabei*	0,12		0,03
	3 *Paracobanocythere grandis*	0,26	0,21	

## Discussion

*Paracobanocythere vietnamensis* sp. n. closely resembles *Paracobanocythere watanabei* and *Paracobanocythere hawaiiensis* according to the appendage morphology, including chaetotaxy and shapes of the podomeres; however, they have divergent male copulatory organ morphologies (Table 3). Some interstitial ostracods, e.g., species from the genera *Microloxoconcha* and *Parapolycope*, are distinguished by specific differences only in the characters associated with mating or courtship; these are not highly divergent in carapace morphology, and the other appendages have almost no differences (see Higashi and Tsukagoshi 2008, Tanaka and Tsukagoshi 2013). Therefore, most diagnostic characters appear in reproductive features such as the male copulatory organ, which can be used to identify interstitial species rather than surface-dwelling ostracods. This is possibly due to the simplification of appendage morphologies driven by the adaptation to the interstitial environment (Hartmann 1973, Maddocks 1976, Danielopol 1976) and the relatively large size of reproductive characters such as the male copulatory organ (Polilov and Beutel 2009). The *Paracobanocythere* species are likely not an exception to this observed taxonomic tendency.

We discovered that the evolutionary distances among three Asian species of *Paracobanocythere* ranged from 0.37±0.03 to 0.41±0.03 according to the nucleotide sequences (Table 4). This value is almost identical to the interspecies genetic distances of other podocopid ostracods (Yamaguchi 2000, Higashi et al. 2011) or somewhat larger (Martens et al. 2005, Brandăo et al. 2010, Karanovic 2015). The distance revealed by the nucleotide and amino acid sequences demonstrated that the distance between *Paracobanocythere vietnamensis* sp. n. and *Paracobanocythere watanabei* is smaller than that between *Paracobanocythere vietnamensis* sp. n. and *Paracobanocythere grandis* or *Paracobanocythere watanabei* and *Paracobanocythere grandis* (Table 4). It is highly possible that *Paracobanocythere vietnamensis* sp. n. and *Paracobanocythere watanabei* are phylogenetically closely related. The similar carapace and appendage morphologies (see Table 3) and small evolutionary distance between these lineages also supports this suggestion. In the future, the discoveries of additional undescribed species and molecular phylogenetic analyses with summarizing their fossil records will shed light on the evolutionary story of the interstitial genus *Paracobanocythere*.

This new species is the first marine interstitial ostracods described from Southeast Asia. Since there have been no taxonomic studies of interstitial ostracods in this region, their biodiversity has largely remained unknown. The Southeast Asian region (the Oriental realm) is known as a marine biodiversity hotspot (see Roberts et al. 2002), likely harboring an abundance of undescribed species. Therefore, we can expect that there are also highly diverse interstitial ostracods in the region since the majority of interstitial genera are distributed globally (Rao 1972, Hartmann 1973, Gottwald 1983, Higashi and Tsukagoshi 2008). Finally, we suggest that more intensive studies are needed in this area, which could reveal cryptic diversity of interstitial ostracods in this underexplored geographic location.

## Supplementary Material

XML Treatment for Paracobanocythere vietnamensis
